# The role of intra-operative parathyroid hormone assay in non-localized adenoma

**DOI:** 10.12669/pjms.38.8.5688

**Published:** 2022

**Authors:** Noor Ezmas M, Norasyikin AW, Nani ML

**Affiliations:** 1 Dr. Noor Ezmas Mahno, MD. Clinical Fellow, Department of Surgery, Clinical Lecturer, Department of Surgery, Kulliyyah of Medicine, International Islamic University Malaysia, Bandar Indera Mahkota Campus, 25200 Kuantan Pahang, Malaysia; 2 Dr Norasyikin Abd Wahab, MD. Consultant Endocrinologist, Department of Internal Medicine, Faculty of Medicine, Universiti Kebangsaan Malaysia, 56000 Cheras, Kuala Lumpur, Malaysia; 3 Dr Nani Md Latar, MD, PhD. Consultant Breast and Endocrine Surgeon, Department of Surgery, Faculty of Medicine, Universiti Kebangsaan Malaysia, 56000 Cheras, Kuala Lumpur, Malaysia

**Keywords:** Intra-operative, parathyroid hormone, non-localized

## Abstract

The incidence of primary hyperparathyroidism (PHPT) is increasing in trend due to more common practice of routine blood investigations especially in the elderly. Surgery is the only curative therapy in symptomatic patients. We present a case of a 63-year-old lady with generalised body weakness associated with occasional muscle cramps. Her biochemical results were consistent with PHPT. As a result of persistent severe hypercalcemia, surgery was planned. However, the pre-operative anatomical and functional radiological imaging (neck ultrasonography, ^99m^Tc-MIBI and FDG-PET scans) failed to identify the abnormal parathyroid gland. Therefore, bilateral neck exploration with intra-operative parathyroid hormone (io-PTH) measurement was performed. The nodular left thyroid and adenomatous right superior parathyroid glands were removed. Possible causes of negative localization and incorporation of io-PTH in under-resourced countries to ensure successful surgery are discussed.

## INTRODUCTION

Primary hyperparathyroidism (PHPT) is a relatively common disorder arising from an autonomous over-production of parathyroid hormone (PTH) by abnormal parathyroid gland(s). Up to 85% of PHPT involves single gland pathology, while the remaining 15% is due to multi-gland disease (MGD). Patients with symptomatic hypercalcemia will benefit from surgery. Therefore, pre-operative localization plays a crucial role in the management of PHPT, especially in this modern era of minimal access surgery.

Successful localization is expected to avoid unnecessary dissection, shorten the duration of surgery and identify ectopic gland. An extensive body of literature has discussed the accuracy and limitations of various imaging modalities in spot-identification or provision of laterality in PHPT. On the other hand, a non-localized parathyroid gland following several attempts at localization is not common. This poses a significant challenge for the operating surgeon and leads to unnecessary anxiety for patients. Careful planning with administration of intra-operative parathyroid hormone (io-iPTH) measurement may help in achieving successful parathyroidectomy.

## CASE REPORT

A 63-year-old lady with underlying diabetes and hypertension for one year presented with generalized body weakness with muscle cramps for six months. She was found to have an elevated serum calcium level during routine blood investigations. She had a strong family history of malignancy, with oral cancer (uncle), colon cancer (aunt) and vaginal cancer (grandmother). Clinical examination did not reveal any abnormality.

Her corrected serum calcium was 3.0 mmol/L (2.2–2.7 mmol/L), with normal serum phosphate of 0.95 mmol/L (0.74–1.52 mmol/L). The 24-hour urine analysis revealed a normal calcium-to-creatinine clearance ratio. The renal function test, full blood picture, serum and urine electrophoresis were also normal. Her serum intact parathyroid hormone (iPTH) was 12.6 pmol/L (1.7–7.0 pmol/L), consistent with the diagnosis of PHPT.

**Fig.1:**
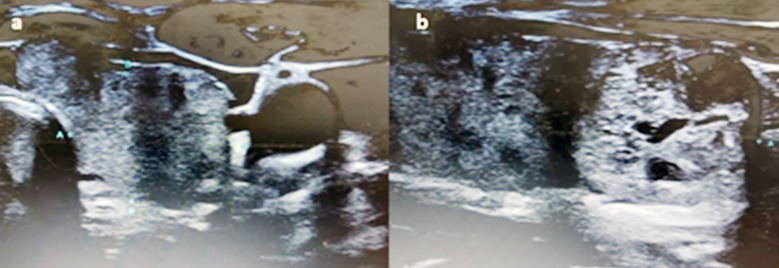
Pre-operative ultrasonography showing the left thyroid lobe on axial view (a) and craniocaudal view (b). No suspicious parathyroid gland detected.

Ultrasonography (USG) of the neck revealed bilateral nodular goiter (Fig.1), with bigger nodules on the left side. No enlarged parathyroid gland(s) were identified. The pre-operative localization imaging investigations using single-isotope ^99m^Technitium-sestamibi (^99m^Tc-MIBI) scanning also revealed negative findings. Therefore, a positron-emission-tomography using fluorodeoxyglucose as radiotracer (FDG-PET/CT) was performed. Unfortunately, this too failed to localize the abnormal parathyroid gland. The skeletal survey was normal and her bone densitometry T-score was -1.0. In view of persistent hypercalcemia with symptomatic bone pain, surgery was warranted.

**Fig.2:**
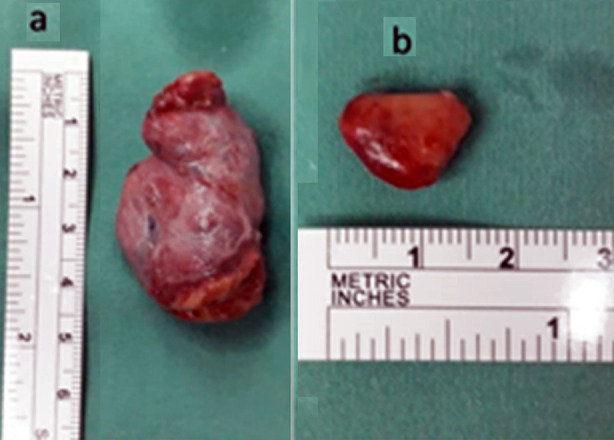
Surgical specimens showing the left thyroid lobe and isthmus (a) and right superior parathyroid adenoma (b).

The patient consented for bilateral neck exploration and left hemi-thyroidectomy. An io-iPTH measurement was arranged as it is not a routine procedure performed in our hospital. The pre-incision iPTH level was 17.0 pmol/L. The left side was explored first, which revealed normal upper and lower parathyroid glands. Upon removal of the left thyroid gland (Fig.2a), the second iPTH sample was taken. Exploration on the right revealed an enlarged superior parathyroid gland measuring 15 × 10 × 5 mm (Fig.2b). Another sample for iPTH was sent following removal of the abnormal gland. Following thyroidectomy, the io-iPTH level dropped to only 13.0 pmol/L, while a significant reduction (>50% from pre-incision) was observed 10 minutes following excision of the right superior parathyroid gland. The final level was 2.8 pmol/L. The patient was discharged with normocalcemic status without calcium supplementation, three days after surgery.

The histopathological report confirmed a right superior parathyroid adenoma weighing 300 mg. The left thyroid lobe was consistent with chronic lymphocytic thyroiditis with an adenomatous nodule.

## DISCUSSION

Pre-operative localization of the abnormal gland in PHPT is required before embarking on a minimal-access surgical approach. However, the potential benefits of this technique must be weighed against the possible disadvantages and cost of multiple imaging investigations, especially in elderly patients with limited allowable radiation exposure.^1^ Various non-invasive imaging investigations have been proposed for localization, including neck USG, ^99m^Tc-MIBI, PET-CT, 4-dimensional computed tomography (4D-CT) and magnetic resonance imaging (MRI). As a guide, a minimum of two concordant investigations with io-iPTH confirmation of complete gland removal are required to ensure successful minimal-access surgery.^1^

A combination of neck USG (anatomical) and ^99m^Tc-MIBI (functional) imaging is commonly instituted due to its cost-effectiveness. A concordant result would improve the accuracy rate from 88 to 96%^2^. However, concordant results occur only in half of these patients.^2^ USG is favored as the first line of investigation due to its low cost and availability as an office-based procedure. Nevertheless, the drawbacks include operator-dependency, presence of thyroid nodules and MGD. To tackle this problem, the sensitivity of USG can be improved with dedicated ultra-sonographer or surgeon-performed USG.^3^ In some cases, USG alone can be efficient to locate the abnormal gland, but ^99m^Tc-MIBI is compulsory for patients with negative USG findings.^3^

^99m^Tc-MIBI is advantageous in localizing deep cervical and ectopic glands. Working on a functional basis, its accuracy may drop from 88% to 44% in detecting a single gland compared to multiple glands.^4^ The presence of thyroid nodules also contributes to false-negative results in ^99m^Tc-MIBI examinations. To dampen this effect, Krishnamurthy et al., demonstrated that the addition of thyroid subtraction may improve its sensitivity.^5^ Therefore, there should be a greater push to standardize parathyroid ^99m^Tc-MIBI protocols, as not all centers routinely performed the scan with thyroid-selective ^99m^Tc-pertechnetate subtraction, as demonstrated in this case.

With the limited resolution of ^99m^Tc-MIBI, negative tests can occur in approximately 20% of patients^5^. Several studies have shown that its accuracy is correlated with the abnormal gland’s weight and size^6^. In our patient, although it was confirmed that she had single gland disease, possible explanations for the negative USG and ^99m^Tc-MIBI may include the small gland size (1.5 cm) and weight (only 300 mg), and the presence of nodular goiter. The two negative imaging prompted clinicians managing this patient to perform the FDG-PET/CT scan. This modality is not routinely performed as the initial pre-operative localization study due to its high cost. However, it has been shown to perform better than ^99m^Tc-MIBI scintigraphy in detecting parathyroid adenoma (87.5% vs 50%) even in the presence of nodular goiter.^7^,^8^ In our patient, however, this combined functional and anatomical imaging failed to identify the enlarged gland. The infrequent usage of this modality might have contributed to the false-negative result.

Non-localized glands pose a lot of anxiety for patients and the attending surgeon due to the uncertain outcome of surgery. Although the success rate of bilateral neck exploration in non-localized PHPT is approaching 95% in good hands^9^, careful planning needs to be instituted in order to minimize morbidity, prevent unnecessarily prolonged surgery, and ensure cure. In some situations, following careful step-by-step exploration with an addition of frozen sections would prove beneficial, especially when routine io-iPTH is not available. In this case, an io-iPTH measurement was arranged to confirm successful removal of the culprit gland.

Io-iPTH required multiple blood samples to be transported immediately on ice. The cost of this procedure must include machine maintenance, calibration, technician and transporter costs, and sample processing. The results are often required urgently to guide decisions on the conclusion of surgery. In our institution, which utilizes the cobas platform for the Roche Analyzer, the patient needed to pay an additional MYR 240 per test (Miami criteria io-iPTH protocol). There are other types of analyzers in the market (e.g., the Beckmann Coulter analyzer) that use a chemiluminescent immunoassay with rapid analysis (less than 15 minutes). This type of machine is expensive, costing MYR 5 million. Therefore, the io-iPTH with almost immediate results is not feasible in our setting. A cost-effectiveness analysis using modelled-predicted outcome scenarios discovered that the cost impact of io-iPTH monitoring depends on institution-specific factors, including the prevalence of MGD^10^. Even though the role of io-iPTH is debatable in cases with concordant preoperative localizations, the capacity of its usage might be different when the culprit gland is not localized preoperatively. For that reason, the higher cost can be offset by the cost of re-operation, invasive re-localization procedures and psychological stress to patients and their family members. In addition, one should consider the additional cost of longer operating time, and greater post-operative pain and morbidity, should further exploration be performed.

It has been documented that the complication and cure rates were similar in non-localized and localized disease.^6^,^9^ However, in the presence of a non-localized gland, good anatomical knowledge of the common, rare and ectopic locations of the parathyroid gland is of utmost importance. The incorporation of io-iPTH in this situation prevents unnecessary dissection and prolongation of surgery. Its usage is commendable, even though it is not a routine procedure performed in our center. When feasible, we should include io-iPTH in bilateral neck exploration procedures, rather than performing additional expensive imaging with more radiation exposure.

During the investigations, the patient and her family were under a lot of stress due to the uncertainties of the surgical outcome. Furthermore, they had to spend a huge sum of money on multiple imaging modalities with inconclusive results. She and her family members were very grateful that surgery had cured her of hypercalcemia.

## CONCLUSION

A non-localized gland poses challenges in the overall surgical management of PHPT. Io-iPTH plays an additional role in bilateral neck exploration to improve surgical success and minimize additional cost related to persistent hypercalcemia.

### Authors’ Contribution:

**NEM, NAW:** Drafted, acquired the data and revised the manuscript.

**NML:** Conceived, drafted, designed and critically revised the manuscript.

All authors have approved the final version and agree to be accountable for all aspects of the work.
